# Physiotherapy for people with mental health problems in Sub-Saharan African countries: a systematic review

**DOI:** 10.1186/s40945-018-0043-2

**Published:** 2018-01-27

**Authors:** Davy Vancampfort, Brendon Stubbs, Michel Probst, James Mugisha

**Affiliations:** 1 Department of Rehabilitation Sciences, KU Leuven – University of Leuven, Tervuursevest 101, 3001 Leuven, Belgium; 2 KU Leuven – University of Leuven, University Psychiatric Center KU Leuven, Leuvensesteenweg 517, 3070 Kortenberg, Belgium; 3 Physiotherapy Department, South London and Maudsley NHS Foundation Trust, London, UK; 4 Health Service and Population Research Department, Institute of Psychiatry, Psychology and Neuroscience, King’s College London, De Crespigny Park, London, UK; 5 Butabika National Referral and Mental Health Hospital, Kampala, Uganda; 6 Kyambogo University, Kampala, Uganda

**Keywords:** Physiotherapy, Physical therapy, Mental health services, Sub-Sahara Africa

## Abstract

**Background:**

There is a need for psychosocial interventions to address the escalating mental health burden in Sub-Saharan Africa (SSA). Physiotherapists could have a central role in reducing the burden and facilitating recovery within the multidisciplinary care of people with mental health problems. The aim of this systematic review was to explore the role of physiotherapists within the current mental health policies of SSA countries and to explore the current research evidence for physiotherapy to improve functional outcomes in people with mental health problems in SSA.

**Methods:**

The Mental Health Atlas and MiNDbank of the World Health Organization were screened for the role of physiotherapy in mental health plans. Next, we systematically searched PubMed from inception until August 1st, 2017 for relevant studies on physiotherapy interventions in people with mental health problems in SSA. The following search strategy was used: “physiotherapy” OR “physical therapy” OR “rehabilitation” AND “mental” OR “depression” OR “psychosis” OR “schizophrenia” OR “bipolar” AND the name of the country.

**Results:**

The current systematic review shows that in 22 screened plans only 2 made reference to the importance of considering physiotherapy within the multidisciplinary treatment. The current evidence (N studies = 3; n participants = 94) shows that aerobic exercise might reduce depression and improve psychological quality of life, self-esteem, body image and emotional stress in people with HIV having mental health problems. In people with depression moderate to high but not light intensity aerobic exercise results in significantly less depressive symptoms (*N* = 1, *n* = 30). Finally, there is evidence for reduction in post-traumatic stress symptoms (avoidance and arousal), anxiety and depression following body awareness related exercises (N = 1, *n* = 26).

**Conclusions:**

Our review demonstrated that physiotherapy is still largely neglected in the mental health care systems of SSA. This is probably due to poor knowledge of the benefits of physiotherapy within mental health care by policymakers, training institutes, and other mental health care professionals in SSA. Based on the current scientific evidence, this paper recommends the adoption of physiotherapy within mental health care services and investment in research and in training of professionals in SSA.

## Background

Mental and substance use disorders are the leading cause of years lived with disability (YLD) in Sub-Saharan Africa (SSA), accounting for almost 20% of all disability-associated burden [1]. The burden is estimated to increase even further with 130% and YLDs will rise from between 20 to 45 million YLDs by 2050 [2] and will be equivalent to about two thirds the YLDs of the entire non-communicable diseases group, which is estimated at 67million YLDs [3]. The consequences of this rising burden of mental and substance use disorders will be devastating. The quality of life of those affected will be impacted and economic costs will be tremendous in these low-resourced settings [4]. Moreover, secondary co-morbidities need to be considered which can add substantively to the increased disability and burden [5, 6]. For example, severe mental illness has been shown to be an independent risk factor for other important non-communicable disorders such as cardio-metabolic diseases, albeit inconsistently in SSA studies [7]. Consistent associations are however reported between HIV/AIDS and chronic pain [8, 9] and between HIV/AIDS and poor mental health [10, 11]. The burden of mental health problems, HIV/AIDS and chronic pain will have larger consequences for women because if their productivity is affected, this directly affects their family welfare and increases the scale of both family and community poverty. Moreover, in SSA, many women are relying on labor-demanding jobs in the informal sector with no job security or compensation for lost income. Maintaining physical strength and an adequate activity level is therefore crucial for their livelihoods.

So far, a mental health policy has been relatively low on the priority list in most of SSA countries, although interest is increasing [12, 13]. However, in most countries still less than 1% of the health budget is spent on mental health [14]. As a result, mental health services are poorly resourced and treatment rates for people with mental disorders remain low, with treatment gaps over 90% [15]. To date, within low-to-middle income countries, community-based rehabilitation, psychoeducation and support for families (delivered by non-specialists) are recommended for low resource settings, with assertive community care and cognitive therapy recommended as additions in higher resourced settings with stronger service-delivery platforms [16].

Since the emphasis in health service delivery in SSA is based on the biomedical model (versus the biopsychosocial model) with an important focus on pharmacology in the management of mental health problems, the potential role of physiotherapy seems to be neglected [13]. As a result, more doctors and nurses are recruited as key cadres as compared to occupational therapists and physiotherapists [13].

There is however worldwide an increasing body of research demonstrating that physiotherapy can improve physical, mental and social health outcomes in people with mental health problems [17, 18]. For example, it has been shown that aerobic and strength exercises and yoga reduce mental health and cognitive symptoms, state anxiety, and psychological distress while it improves health-related quality [17, 18]. Progressive muscle relaxation reduces on its turn state anxiety and psychological distress [17, 18]. Thus, implementation of physiotherapy within the care of people with mental health problems could reduce the mental, physical and social burden, while facilitating functional recovery and consequently reducing disability. This will on its turn reduce the societal costs.

The aim of the current systematic review is twofold. First, we set out to explore the role of physiotherapy within the current mental health policies and plans of SSA countries. Specifically, we wanted to explore which roles were reported and defined for physiotherapists. Second, we explored the current research evidence for physiotherapy in people with mental health problems in Sub-Saharan Africa.

## Methods

### Screening for the role of physiotherapists in mental health policies and plans in sub-Saharan Africa

In a first stage, we screened the latest Mental Health Atlas [19]. If the country data were not available in the latest edition, the penultimate edition was screened. With data from 171 World Health Organization (WHO) Member States, the Mental Health Atlas provides a comprehensive overview of mental health policies, plans and services worldwide. Data abstracted were the presence of a mental health policy and/or plan (yes or no).

In a second stage, if a mental health policy and/or plan was available the full-text documents were retrieved via the MiNDbank of the World Health Organization [20]. Policies written in English, French, Spanish or Portuguese were evaluated. If the mental health policy and/or plan was not available, google scholar was screened using the search terms: “mental health” AND “plan” OR “policy” and the name of the country, or its equivalents in other languages. Mental health policies and plans were screened for the role of physiotherapy. Search terms used, were: “physical therap*” OR “physiotherap*” or its equivalents in other languages.

In a third stage, we summarized the reported role of physiotherapists in the policy plans.

### Identification of physiotherapy studies conducted in people with mental health problems in SSA

#### Search strategy

We systematically search PubMed from inception until August 1st 2017 for relevant studies on physiotherapy in people with mental health problems in SSA. The following search strategy was used: “physiotherapy” OR “physical therapy” OR “rehabilitation” AND “mental” OR “depression” OR “psychosis” OR “schizophrenia” OR “bipolar” AND the name of the country. We did not place a language restriction upon these searches. In addition, the reference lists of all eligible articles were scanned to assess eligibility of additional studies.

#### Eligibility criteria

##### Participants

As we were interested in prevention and treatment of mental health problems, we did not exclude any people due to age or whether or not they were diagnosed with Statistical Manual (DSM) [21, 22], International Classification of Disease (ICD) [23] or with validated diagnostic tools.

##### Interventions

The physiotherapy interventions could comprise aerobic exercises, strength exercises, relaxation training, basic body awareness exercises, or a combination of these in accordance with the World Confederation for Physical Therapy position statement. A physiotherapy intervention could be used alone or in conjunction with other interventions, with physiotherapy being considered the main or active element. Interventions that included physiotherapy in a multiple component weight management program were excluded because the specific effects of the physiotherapy intervention could not be addressed. Other interventions could include any of the following: pharmacotherapy, psycho-education, and cognitive-behavioural or motivational techniques related to changing physical activity behaviour. Excluded were: (a) peer-led physical activity interventions, and (b) mindfulness-based cognitive behavioural therapy.

##### Control conditions

The presence of control conditions was not an inclusion criterion. However, if studies were clinical or randomized controlled, usual-care or wait-list control conditions were included. Usual care, if available, was defined as care that people would normally receive had they not been included in a research trial. Such care would include medication, hospitalization, community support, and outpatient care.

##### Outcome measure

The primary outcome measure was any mental or social health outcome measures. Secondary outcomes were physical health outcomes in mentally ill populations.

##### Study design

We included pre- and post-test studies without a control group and randomized (RCTs) or non-randomized clinical controlled trials (NRCCTs) in which the experimental and control intervention were of similar duration. Qualitative and quantitative data were included.

##### Exclusion criteria

In case of overlap only the most recent study or the study with the largest dataset were included. No additional exclusion criteria were applied.

### Study selection

Two reviewers (DV and BS) screened titles and abstracts of all potentially eligible articles. Both authors applied eligibility criteria, and a list of relevant studies was developed through consensus. When necessary, the protocol stated that the corresponding author of a study would be contacted up to two times in a 4-week period to request data that would enable inclusion in the current analyses.

### Data extraction

Two authors (DV, BS) extracted data using a predetermined data extraction form. The data extracted for were country, study setting, patient characteristics (diagnosis, age, % male). Duration (weeks) frequency (times per week), intensity (as defined by the authors), and type (aerobic exercise, resistance training, relaxation training, basic body awareness exercises or a mixed combination) of the physiotherapy intervention. Finally, we did extract any relevant outcomes and motivational strategies used to improve adherence and reduce dropout from the physiotherapy interventions.

### Methodological quality assessment of the RCTs

Two authors (DV and BS) assessed studies on the presence of high, low risk or unclear risk of bias according to the Cochrane Handbook definition [24]. Studies presenting adequate allocation concealment and complete presentation of outcome data (intention-to-treat analysis) and blinding outcome assessors are considered studies with low risk of bias (high quality trials).

## Results

### Physiotherapy priorities in mental health policies and plans in sub-Saharan Africa

In terms of policy, 69% (=33/48) of SSA countries report having a mental health policy or plan. Ten policies were not found while one (Sudan) was written in Arabic, and therefore not meeting our inclusion criteria and was not screened. Two of 22 screened mental health policies or plans included a physiotherapy component. An overview of the presence of a mental health policy or plan and the presence of a physiotherapy component is presented in Table 1. The roles described in the mental health policies of Namibia and Nigeria are prescribed in Table 2. Briefly, while physiotherapists were only described as member of the multidisciplinary team in Nigeria, in Namibia a more extensive role was defined including facilitating the integration of mental health in primary health care, identifying training needs and assisting in public mental health campaigns.

**Table 1:** Overview of the presence of a mental health policy/plan, whether there was a role for physiotherapists and physiotherapy research results in Sub-Saharan African countries (*n* = 48)

Country	Official mental health policy or plan	Role for physiotherapists	PubMed search results (potential relevant / obtained)
Angola (2011)	Yes	No	0/10
Benin (2014)	No	/	0/65
Botswana (2014)	Yes	No	0/38
Burkina Faso (2014)	Yes	No	0/25
Burundi (2014)	Yes	No	0/14
Cameroon (2011)	No	/	0/48
Cape Verde (2011)	Yes	No	0/6
Central African Republic (2014)	Yes	No	0/4
Chad (2011)	Yes	NA	0/37
Comoros (2011)	Yes	NA	0/5
Congo (2014)	No	/	0/76
Côte d’Ivoire (2014)	Yes	No	0/21
Democratic Rep. of the Congo (2011)	Yes	No	0/36
Djibouti (2014)	No	/	0/6
Equatorial Guinea (2014)	No	/	0/0
Eritrea (2011)	No	/	0/19
Ethiopia (2014)	Yes	No	0/277
Gabon (2011)	No	/	0/1
Gambia (2014)	Yes	No	0/17
Ghana (2014)	Yes	No	1/127
Guinea (2014)	Yes	NA	0/3151
Guinea-Bissau (2011)	No	/	0/0
Kenya (2011)	Yes	No	0/207
Lesotho (2014)	No	/	0/7
Liberia (2014)	Yes	No	0/14
Madagascar (2014)	Yes	No	0/12
Malawi (2014)	Yes	No	0/59
Mauritania (2011)	Yes	NA	0/7
Mauritius (2014)	No	/	0/16
Mozambique (2014)	Yes	No	0/13
Namibia (2014)	Yes	Yes	0/16
Niger (2011)	Yes	NA	0/94
Nigeria (2014)	Yes	Yes	5/878
Rwanda (2014)	Yes	No	1/65
São Tomé and Príncipe (2014)	Yes	NA	0/0
Senegal (2014)	No	/	0/50
Seychelles (2014)	No	/	0/9
Sierra Leone (2014)	Yes	No	1/16
Somalia (2014)	No	/	0/54
South-Africa (2014)	Yes	No	6/1632
South-Sudan (2014)	No	/	0/7
Sudan (2011)	Yes	Not checked^a^	0/89
Swaziland (2014)	No	/	0/13
Togo (2014)	Yes	NA	0/55
Uganda (2014)	Yes	No	2/294
United Republic of Tanzania (2011)	Yes	NA	0/123
Zambia (2014)	Yes	No	0/44
Zimbabwe (2014)	Yes	NA	0/102
Summary	69% (33/48) has an official mental health policy	% (2/22) described a role for physiotherapists	16/7859

*UN* Unknown, *NA* Not available

^a^plan written in Arabic

**Table 2:** The role of physiotherapists in mental health plans of Sub-Saharan African countries

Country	Role of physiotherapists
Namibia	Physiotherapists should: -facilitate the integration of the mental health policy into primary health care -identify specific training needs in mental health care -provide input into the development of (the World Health Organization affiliated) Information, Education and Communication materials, policies, guidelines and standards for mental health
Nigeria	Physiotherapists are involved in mental health care services, but a specific role was not defined.

### Screening for physiotherapy studies conducted in people with mental health problems in SSA

#### Search results

Out of 7859 search hits, 16 potentially eligible studies were retrieved. After applying the eligibility criteria 5 [25–29] studies were included. An overview of the search results for each country is presented in Table 1. Reasons for exclusion were: not limited to SSA countries (*n* = 1), peer-led interventions (n = 1), no relevant outcomes (*n* = 2), sport-for-development by community members with no input from physiotherapists (n = 2), mindfulness based cognitive behavioral therapy (n = 2), and cross-sectional studies on physical activity (*n* = 3).

#### Participants and study characteristics

Four RCTs [25–28] and one qualitative study [29] involving in total 241 participants were included. Three RCTs [25, 27, 28] focused on mental health outcomes in people with HIV and one focused on mental health outcomes in people with depression [26]. All RCTs explored aerobic exercise. The qualitative study explored the effect of dance movement and body awareness related exercises in torture survivors and former child soldiers (Sierra Leone). The interventions ranged from 6 to 26 weeks, from 30 to 90 min per week, from 2 to 3 times per week and from low to high intensity. Details of the participants and study characteristics are presented in Table 3.

**Table 3:** Mental and/or physical health outcomes in physiotherapy related studies in Sub-Saharan Africa

First author	Country	Design	Participants	Physiotherapy intervention	Mental and/or physical health outcomes*	MQ
Aweto 2016	Nigeria	RCT	18 (32.1 ± 5.4 years) outpatients with HIV; BMI = 26.1 ± 1.4 vs 15 controls with HIV with care as usual (30.7 ± 5.8 years); 10♂/33	6 weeks, 3*week, 30 min moderate intensity aerobic exercise on a cycle ergometer provided by a physiotherapist	The Beck Depression Index score only reduced significantly in the exercise group [10.3 ± 6.5 vs.3.5 ± 1.3;P < 0.001]	
Balchin 2016	South-Africa	RCT	30♂ moderately depressed; mean age = 25.4 years, mean BMI = 26.9	6 weeks, 3*week, 60 min high vs moderate vs low intensity aerobic exercise; providers unknown	The HAM-D (15.9 ± 1.8 vs. 5.7 ± 5.8 and 16.4 ± 1.4 vs. 6.6 ± 5.0 vs. 17.1 ± 1.2 vs. 11.8 ± 3.9, respectively) and MADRS 12.7 ± 4.0 vs. 7.0 ± 6.7 and 14.4 ± 4.3 vs. 9.0 ± 6.7 vs. 18.8 ± 6.4 vs. 15.0 ± 5.2, respectively) only reduced significantly in the high and moderate intensity aerobic exercise	+
Maharaj 2011	South-Africa	RCT	26 (16♂) (19–58 years) outpatients on antiviral therapy vs 26 (18♂) (22–51 years)	weekly aerobic exercise on cycle ergometer (2*10 min) and treadmill (2*10 min) at 50–70% of the age predicted maximum heart rate for 3 months	Significant improvements in all SF-36 domains (*P* < 0.05) for the experimental group compared with the control group, with the physical SF-36 summary scores (*P* < 0.018) and mental SF-36 summary scores (*P* < 0.021) scores being significantly higher after exercise.	+
Mutimura 2008	Rwanda	RCT	50 (20♂) (37.5 ± 6.9 years) outpatients with HIV; 88% employed; BMI = 24.4 ± 2.7; 20% smoking vs 50 (20♂) controls with HIV with care as usual (37.8 ± 5.5 years)	26 weeks, 3*week, 90 min moderate intensity aerobic and resistance training; providers unknown	At 6 months, scores on psychological quality of life [1.3 ± 0.3 vs. 0.5 ± 0.1; *P* < 0.0001], self-esteem [1.3 ± 0.8 vs. 0.1 ± 0.6); *P* < 0.001], body image [1.5 ± 0.6 vs. 0.0 ± 0.5; *P* < 0.001] and emotional stress [1.6 ± 0.7 vs. 0.2 ± 0.5; P < 0.001], improved more in the exercise group	
Harris 2007	Sierra Leone	Qualitative	Three studies: [1] 6♀ (16–17 years), [2] 8♂ (23–24 years), [3] 12♂ (8 aged 18) child soldiers and torture survivors	9 to 16 weekly sessions of dance movement therapy with body awareness exercises within psychotherapy	Reduction in self-reported post-traumatic stress symptoms (avoidance and arousal), anxiety and depression	NA

*RCT* Randomized controlled trial, *HAM-D* Hamilton depression score, *MADRS* Montgomery-Åsberg Depression Rating Scale, *SF-36* Health Related Quality of Life Short Form – 36. *MQ* Methodological quality: risk of bias was assessed on random sequence generation, allocation concealment, blinding of participants, blinding of those delivering the intervention, blinding of outcome assessors, incomplete data outcome, selective reporting or others. Studies presenting adequate allocation concealment and complete presentation of outcome data (intention-to-treat analysis) and blinding outcome assessors are considered studies with low risk of bias (high quality trials, coded with “+”); NA = not applicable (no RCT)

### Methodological quality of RCTs

Two of the four RCTs were considered to be of high methodological quality with low risk of bias (see Table 3). The most important risks increasing bias in the other RCTs were lack of intention-to-treat analyses or a lack of blinding of the assessors.

#### Physiotherapy outcomes

In the 3 RCTs [25, 27, 28] in people with HIV aerobic exercise reduced depression and improved psychological quality of life, self-esteem, body image and emotional stress. In one RCT [26] in depressed adults only moderate and high but not light intensity aerobic exercise resulted in significantly less depressive symptoms. In the qualitative study [29] a reduction in post-traumatic stress symptoms (avoidance and arousal), anxiety and depression were reported following body awareness related exercises. Details of the physiotherapy intervention characteristics and outcomes are presented in Table 3.

## Discussion

The current systematic review shows that in those SSA countries that have a mental health policy or plan (70%) only two make reference to a role for physiotherapy. Therefore, although physiotherapy is becoming acknowledged as an important adjunct in the management of mental health problems [30, 31], the potential is yet to be embraced in SSA countries. The lack of priority given to physiotherapy as a complementary treatment is also mirrored in the limited number of studies exploring the importance of physiotherapy interventions in the management of mental health problems. Five studies, of which 4 RCTs, were found indicating that aerobic exercise can improve mental health outcomes while there is qualitative evidence from one study for improvements in quality of life following body awareness related exercises.

It is plausible that due to the strong biomedical focus on pharmacotherapy [13] health care professionals in SSA are yet to be fully aware of the psychosocial effects of physiotherapy. Hence, a need to re-orient the current health care systems including policy makers to embrace physiotherapy in the management of mental health problems is needed. Budget holders should also invest more in mental health research. In three of the five studies mental health outcomes were explored in people with HIV. This is not surprising and due to increased funding of HIV/AIDS care and research on the continent. Mental health research and in particular research exploring the role physiotherapists could benefit current mental health care systems. Future physiotherapy studies in different contexts in SSA could explore:

- whether individually prescribed physiotherapy can improve mood, reduce stress, promote well-being and address somatic co-morbidities associated with mental health diagnoses in inpatients, outpatients and community patients.
- whether treatment of physical problems and physical pain of people with mental health diagnoses by physiotherapists will facilitate social participation and recovery.
- whether physiotherapy interventions can minimise the metabolic and motor side-effects of some psychotropic medications.
- the benefits of fall prevention for elderly with mental health problems or for those on antipsychotic medication.
- whether physiotherapy could improve self-management and coping strategies in the context of mental and physical health issues.
- whether body awareness exercise can boost a person’s self-esteem.

Within mental health care systems, physiotherapists should be seen as valuable members of a multidisciplinary approach. Several strategies to initiate and stimulate physiotherapy within the mental health care systems of SSA are possible. For example, continued medical education, which is a common practice in SSA [13], should be used to inform supervising mental health care professionals on the importance of physiotherapy. Policy makers should be made aware that investment in physiotherapy could optimize mental and physical health improvements while inclusion of physiotherapists in lifestyle interventions will improve adherence and reduce dropout [32, 33] and consequently will be cost-effective. Physiotherapy institutions should focus on the importance of physiotherapy for mental health in order to improve the competencies of their graduates in this field. All physiotherapists should be able to recognize the signs and symptoms of the main disorders and demonstrate basic knowledge of the causes. Next to this, physiotherapists should be trained in basic therapeutic interactions (e.g. communication, attitude) and in basic motivational skills (e.g., motivational interviewing). Research should explore the most optimal form of physiotherapy delivery in different contexts in low-resourced settings. For example, physiotherapy trials in different settings could explore whether assisting people with mental health problems in fulfilling three universal, psychological needs: (a) the need for autonomy (i.e., experiencing a sense of psychological freedom when engaging in physiotherapy exercises), (b) the need for competence (i.e., ability to attain desired outcomes following physiotherapy), and (c) the need for relatedness (i.e., feeling socially connected) will increase the likelihood that they adopt or maintain for example an active lifestyle [34].

The current findings should be interpreted in light of some methodological limitations. First of all, as we only searched PubMed, several relevant articles might be missed. However, to minimize this risk, we performed additional hand search by reviewing references listed in the included original publications. Second, due to the limited literature available we were not able to perform a more rigorous meta-analysis.

## Conclusions

The current data shows that in SSA the importance of considering physiotherapy in mental health care is largely ignored in the current policies and within the current research. Policy makers and existing work force should be informed about the potential of physiotherapy in the multidisciplinary treatment of people with mental health problems. There is a need to invest in physiotherapy in the mental health care settings, and research and training in institutes of SSA countries. There is already, however limited, evidence that the inclusion of physiotherapists in the treatment will improve outcomes.

## Abbreviations

SSA: Sub-Saharan African
YLD: Years lived with disability
